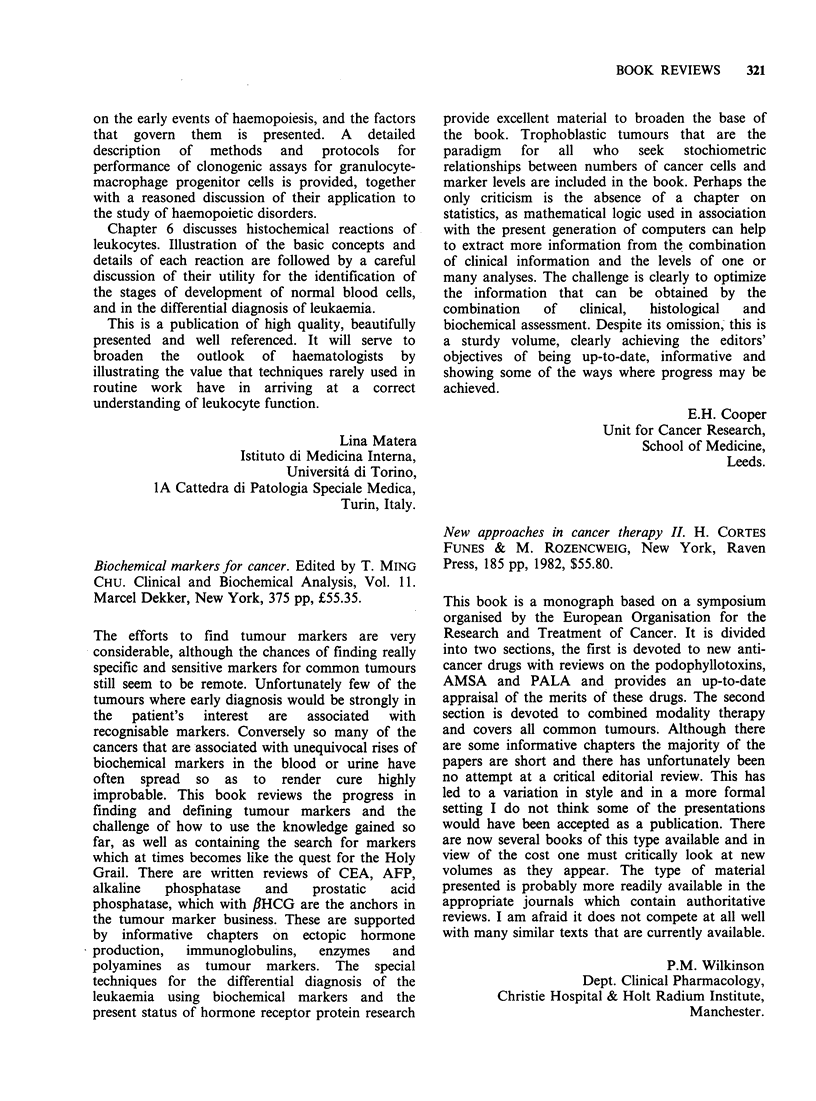# Biochemical markers for cancer

**Published:** 1983-02

**Authors:** E.H. Cooper


*Biochemical markers for cancer*. Edited by T. MING CHU. Clinical and Biochemical Analysis, Vol. 11. Marcel Dekker, New York, 375 pp, £55.35.

The efforts to find tumour markers are very considerable, although the chances of finding really specific and sensitive markers for common tumours still seem to be remote. Unfortunately few of the tumours where early diagnosis would be strongly in the patient's interest are associated with recognisable markers. Conversely so many of the cancers that are associated with unequivocal rises of biochemical markers in the blood or urine have often spread so as to render cure highly improbable. This book reviews the progress in finding and defining tumour markers and the challenge of how to use the knowledge gained so far, as well as containing the search for markers which at times becomes like the quest for the Holy Grail. There are written reviews of CEA, AFP, alkaline phosphatase and prostatic acid phosphatase, which with βHCG are the anchors in the tumour marker business. These are supported by informative chapters on ectopic hormone production, immunoglobulins, enzymes and polyamines as tumour markers. The special techniques for the differential diagnosis of the leukaemia using biochemical markers and the present status of hormone receptor protein research provide excellent material to broaden the base of the book. Trophoblastic tumours that are the paradigm for all who seek stochiometric relationships between numbers of cancer cells and marker levels are included in the book. Perhaps the only criticism is the absence of a chapter on statistics, as mathematical logic used in association with the present generation of computers can help to extract more information from the combination of clinical information and the levels of one or many analyses. The challenge is clearly to optimize the information that can be obtained by the combination of clinical, histological and biochemical assessment. Despite its omission, this is a sturdy volume, clearly achieving the editors' objectives of being up-to-date, informative and showing some of the ways where progress may be achieved.

E.H. Cooper
Unit for Cancer Research,
School of Medicine,
Leeds.